# Encapsidation of Viral RNA in *Picornavirales*: Studies on Cowpea Mosaic Virus Demonstrate Dependence on Viral Replication

**DOI:** 10.1128/JVI.01520-18

**Published:** 2019-01-04

**Authors:** Inga Kruse, Hadrien Peyret, Pooja Saxena, George P. Lomonossoff

**Affiliations:** a Department of Biological Chemistry, John Innes Centre, Norwich, United Kingdom; University of Maryland, College Park

**Keywords:** *Picornavirales*, RNA packaging, RNA virus, cowpea mosaic virus, plant viruses, viral encapsidation, viral replication

## Abstract

To elucidate the linkage between replication and encapsidation in *Picornavirales*, we have taken advantage of the bipartite nature of a plant-infecting member of this order, cowpea mosaic virus (CPMV), to decouple the two processes. RNA-free virus-like particles (empty virus-like particles [eVLPs]) can be generated by transiently coexpressing the RNA-2-encoded coat protein precursor (VP60) with the RNA-1-encoded 24,000-molecular-weight (24K) protease, in the absence of the replication machinery (K. Saunders, F. Sainsbury, and G. P. Lomonossoff, Virology 393:329–337, 2009, https://doi.org/10.1016/j.virol.2009.08.023). We have made use of the ability to produce assembled capsids of CPMV in the absence of replication to examine the putative linkage between RNA replication and packaging in the *Picornavirales*. We have created a series of mutant RNA-1 and RNA-2 molecules and have assessed the effects of the mutations on both the replication and packaging of the viral RNAs. We demonstrate that mutations that affect replication have a concomitant impact on encapsidation and that RNA-1-mediated replication is required for encapsidation of both RNA-1 and RNA-2. This close coupling between replication and encapsidation provides a means for the specific packaging of viral RNAs. Moreover, we demonstrate that this feature of CPMV can be used to specifically encapsidate custom RNA by placing a sequence of choice between the RNA-2 sequences required for replication.

**IMPORTANCE** The mechanism whereby members of the order *Picornavirales* specifically package their genomic RNAs is poorly understood. Research with monopartite members of the order, such as poliovirus, indicated that packaging is linked to replication, although the presence of “packaging signals” along the length of the viral RNA has also been suggested. Thanks to the bipartite nature of the CPMV genome, which allows the manipulation of RNA-1 without modifying RNA-2, we show here that this specificity is due to a functional link between the two processes of viral replication and encapsidation. This has important implications for our understanding of the fundamental molecular biology of *Picornavirales* and opens the door to novel research and therapeutic applications in the field of custom RNA packaging and delivery technologies.

## Abstract (precis)

The mechanism whereby members of the order *Picornavirales* specifically package their genomic RNAs is poorly understood. Research with monopartite members of the order, such as poliovirus, indicated that packaging is linked to replication, although the presence of “packaging signals” along the length of the viral RNA has also been suggested. Thanks to the bipartite nature of the CPMV genome, which allows the manipulation of RNA-1 without modifying RNA-2, we show here that this specificity is due to a functional link between the two processes of viral replication and encapsidation. This has important implications for our understanding of the fundamental molecular biology of *Picornavirales* and opens the door to novel research and therapeutic applications in the field of custom RNA packaging and delivery technologies.

## INTRODUCTION

The *Picornavirales* are a large order of single-stranded positive-sense viruses that form icosahedral particles. They have genomes consisting of either one (e.g., poliovirus, foot-and-mouth disease virus, and hepatitis A virus) or two (cowpea mosaic virus [CPMV]) strands of positive-sense, single-stranded RNA that are encapsidated within protein capsids of 28 to 30 nm in diameter. The viral capsids exhibit pseudo-T=3 (P=3) icosahedral symmetry, comprising 60 asymmetric units, each with 3 distinct β-barrel domains. These domains can either remain as a single polypeptide or be processed to give two or three distinct coat protein subtypes; in some cases, additional processing occurs to generate small proteins which are found within capsids. Because of their importance as pathogens and their relatively simple, nonenveloped capsids, structures of the *Picornavirales* have been extensively studied by both X-ray crystallography and cryo-electron microscopy (1). Such studies have revealed the structure of the coat proteins in great detail and are beginning to provide some information about the structure adopted by the RNA within the particles (2–4).

In contrast to this wealth of structural detail, little is known about the features that control the specificity of RNA encapsidation. Viral particles contain almost exclusively their cognate genome (5–7), implying that there must be an efficient mechanism for discriminating between host and viral sequences. Analysis of the genomes of members of the order *Picornavirales* has not revealed the presence of any extensive sequences on the genomic RNA that could act as “packaging signals” (5), although recent work on the binding of aptamers to partially disassembled parechovirus capsids suggested that multiple short stretches of sequences within the genome affect the efficiency of packaging (8). There is, however, some evidence that packaging of genomic RNA is coupled to viral replication in poliovirus (6, 9), and replication-associated proteins have been found to be associated with capsids in poliovirus and foot-and-mouth disease virus (10). If only RNAs replicated from an RNA template were capable of being packaged, this would provide a means of distinguishing viral from cellular RNAs. However, definitive proof of the coupling between replication and encapsidation is lacking; to date, research in this area has been carried out on monopartite viruses, in which it is very difficult to study replication and encapsidation independently.

**FIG 1:**
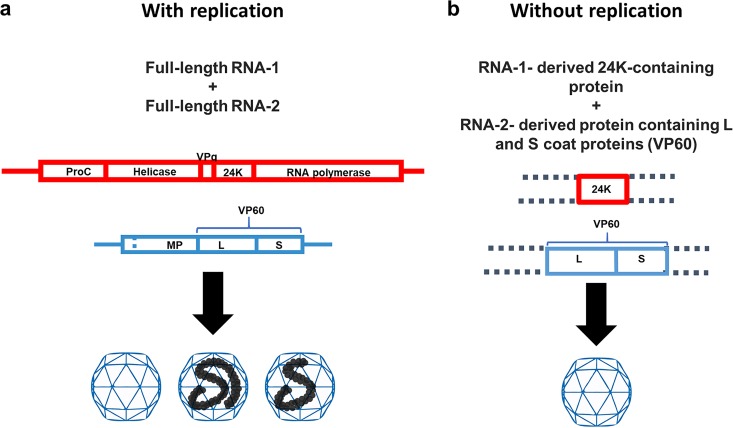
The bipartite CPMV replication and packaging system. (a) When full-length, replication-competent RNA-1 and RNA-2 are coexpressed, three species of particles are formed: empty (top component), RNA-2-containing (middle component), and RNA-1-containing (bottom component) species. (b) The coexpression of any 24K protease-containing sequence with any VP60-containing sequence in the absence of viral replication will yield empty particles indistinguishable from the top component (4, 23).

CPMV is the type member of the comoviruses, a plant-infecting genus within the *Picornavirales*, that has a genome consisting of two, separately encapsidated RNA molecules (RNA-1 and RNA-2). As a result, infections with CPMV produce three types of particles with identical protein compositions: top (T), middle (M), and bottom (B). These particles contain, respectively, no RNA, exclusively RNA-2, and exclusively RNA-1 (Fig. 1a). Both RNA-1 and RNA-2 are translated into polyproteins that are processed by the RNA-1-encoded 24,000-molecular-weight (24K) protease. RNA-1 encodes all the functions necessary for viral replication and can self-replicate in individual plant cells, as seen in studies using plant protoplasts (11). RNA-2 encodes the precursor (VP60) for the two viral coat proteins, large (L) and small (S), as well as the 48K protein involved in cell-to-cell movement of the virus (12). RNA-2 is dependent on the RNA-1-encoded proteins for its replication and for cleavage of the coat protein precursor. The natural separation of the replicative and structural functions on different RNAs in CPMV makes the virus an attractive system for studying the possible linkage between replication and encapsidation in *Picornavirales*.

An advantage of using a plant-based system for examining the potential link between replication and encapsidation is the availability of a highly efficient agrobacterium-mediated transient-expression system, the pEAQ vector system, that enables the expression of proteins in leaves in the absence of viral RNA replication (13, 14). This system has been used to express a wide variety of proteins and protein complexes, including virus-like particles (VLPs), in plants (15–21). In the case of members of the *Picornavirales*, CPMV and poliovirus (22, 23), the generation of VLPs requires the coexpression of both a coat protein precursor and the cognate protease needed for its processing. The coexpression of these components in the absence of RNA replication results in the production of large quantities of RNA-free VLPs in which the assembled protein shell is identical in structure to the natural, RNA-containing capsids (2, 4, 22, 24, 25) (Fig. 1b). The lack of any detectable RNA within CPMV or poliovirus VLPs (2, 21, 22) contrasts with the situation found with many other plant-expressed VLPs that, in the absence of replicating RNA, package host-derived RNAs (18, 26). This is consistent with these viruses having a specific mechanism for selecting only virus-derived RNAs for packaging. In the case of CPMV, when a replication-competent version of RNA-1 was used as a source of the 24K protease, it was specifically encapsidated into CPMV-like particles (23), demonstrating the CPMV capsids produced via transient expression are capable of encapsidating viral RNA. However, whether the specific encapsidation of RNA-1 was due to its ability to self-replicate or was a feature of its length and sequence, such as the presence of packaging signals, was not investigated.

In this study, we make use of the availability of a system for making capsids via transient expression in the absence of RNA replication to investigate the linkage between replication and encapsidation in the *Picornavirales*. This involves the expression of empty CPMV VLPs (eVLPs) via the transient expression of the CPMV coat protein precursor VP60 and RNA-1-derived proteins containing the CPMV 24K protease domain needed for its correct processing (23). By coexpressing versions of CPMV RNA-1 and RNA-2 with different replication efficiencies, it is possible to examine the relationship between replication and encapsidation efficiency (Fig. 1). Using this approach, we have established a clear correlation between the replication competence and encapsidation efficiency of the viral RNAs and show that modified RNA-2 molecules can be packaged, provided that they are replication-competent. These findings are likely to be applicable to all members of the order, whether mono- or bipartite.

## RESULTS

### Packaging of CPMV RNAs requires the presence of a functional RNA-1 molecule.

**FIG 2:**
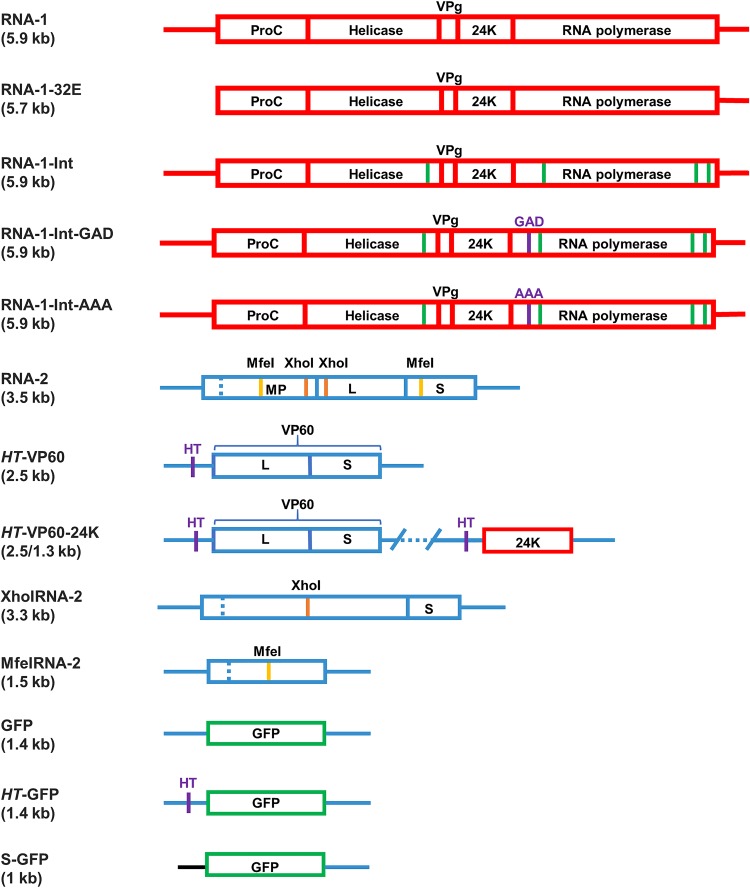
Diagrams of constructs used in this study. Boxes represent open reading frames, and lines represent UTRs. (Top) RNA-1-based constructs (red). Green lines represent introns introduced into the RNA-1 sequence to alleviate toxicity of the DNA constructs in bacteria. Point mutations of the replicase GDD motif are shown in purple, and the amino acid sequence that replaces GDD is indicated. (Bottom) RNA-2-based constructs (blue). The *HT* mutations shown in the 5′ UTR of the *HT*-VP60 construct are a pair of point mutations that greatly increase the translational efficiency of the downstream open reading frame (49). XhoI-RNA-2 and MfeI-RNA-2 are truncated by restriction digestion with the respective enzymes and subsequent religation. The GFP substitution is shown in green, and the synthetic 5′ UTR substituted for the 5′ UTR of RNA-2 in the S-GFP construct is shown in black. All constructs expressed from a pEAQ plasmid also contain a P19 silencing suppressor cassette on the same T-DNA (not shown). The constructs expressed from pBIN plasmids (RNA-1 from pBinPS1NT, RNA-1-32E from pBinPS32E, and RNA-2 from pBinPS2NT) were coinfiltrated with pBIN61-P19 to supply P19. All constructs are expressed in plants transiently via *Agrobacterium*-mediated expression.

To determine whether the ability of wild-type (wt) RNA-1 to be encapsidated in coexpressed CPMV VLPs is due to its ability to self-replicate, plasmids encoding two different versions of CPMV RNA-1 were utilized: pBinPS1NT, containing an infectious full-length version of RNA-1 (27), and pBinP32E (28), containing a mutant RNA-1 (RNA-1-32E) which lacks the 5′ untranslated region (UTR) (Fig. 2) and has previously been shown to no longer self-replicate or support the replication of RNA-2 (28). However, the open reading frame of RNA-1-32E is intact, and it can be translated to give an RNA-1-derived polyprotein that contains a functional 24K protease domain.

To provide a source of the coat proteins necessary for encapsidation, each of the RNA-1 constructs was coinfiltrated into Nicotiana benthamiana leaves with either pEAQ-*HT*-VP60, a construct that encodes both the precursor of the L and S proteins (VP60) and the P19 suppressor of silencing (23), or a mixture of pBinPS2NT, which contains a full-length copy of RNA-2 (27), and pBIN61-P19 to supply P19. In cases where RNA-1 is present, whether or not it is capable of replication, the processing of the VP60 precursor to the mature L and S proteins, which is essential for VLP formation (23), occurs via the 24K protease domain within the RNA-1-encoded polyprotein, (23). As a negative control, leaves were infiltrated with pEAQ-*HT*-VP60 alone.

**FIG 3:**
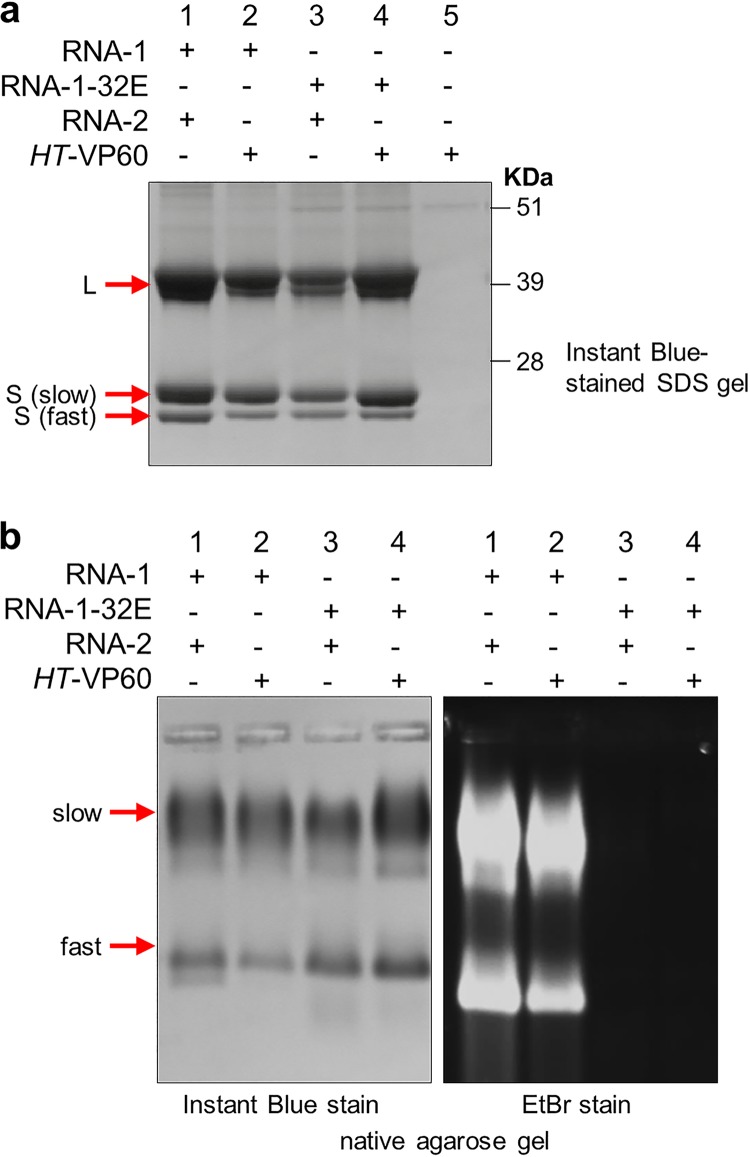
A replication-competent version of RNA-1 is necessary for RNA encapsidation. CPMV particles were purified from N. benthamiana leaves agroinfiltrated with pBinPS1NT and pBinPS2NT (lanes 1 in each gel), pBinPS1NT and pEAQ-*HT*-VP60 (lanes 2), pBinP32E and pBinPS2NT (lanes 3), and pBinP32E and pEAQ-*HT*-VP60 (lanes 4) or with pEAQ-*HT*-VP60 alone (lanes 5). In each case, the CPMV RNAs expressed within the leaves are indicated. The purified particles were examined by either denaturing SDS-PAGE followed by staining with Instant Blue (a) or electrophoresis on a nondenaturing agarose gel (b) followed by staining with either Instant Blue (left) to visualize protein or ethidium bromide (EtBr) (right) to visualize nucleic acid. The positions of the large (L) coat protein and two forms of the small [S (slow) and S (fast)] coat protein are indicated to the left of the gel in panel a. Note that in panel b, CPMV particles separate into distinct electrophoretic populations based on the presence or absence of the labile 24 amino acids at the C terminus of the small coat protein (2) as seen in panel a.

Six days after infiltration, any particles present in the agroinfiltrated leaves were extracted and purified using a method which is known to be highly efficient in producing quantities of both RNA-containing particles and eVLPs (29) that are sufficiently pure for structural studies (2, 4, 24, 25). Transmission electron microscopy (TEM) of negatively stained particle preparations revealed the presence of large quantities of typical CPMV-like particles in all preparations except that from leaves infiltrated with pEAQ-*HT*-VP60 alone; this was expected since uncleaved VP60 cannot form particles (23). SDS-PAGE analysis followed by staining with Instant Blue (Fig. 3a) revealed the presence of the mature L and S proteins in all preparations, apart from that from leaves infiltrated with pEAQ-*HT*-VP60 alone (lane 5), with negligible levels of contaminating host proteins being detected. In each case, the S coat protein is resolved into 2 bands as a result of the presence or absence of the labile C terminus (25). The particle yield was estimated to be in the range of 0.4 to 0.5 g/kg fresh weight tissue for all four samples where a version of RNA-1 was supplied (Fig. 3, lanes 1 to 4), based on the intensities of bands on the gel. The fact that similar yields of the L and S coat proteins were obtained in all four samples provides evidence that in the absence of replication, the coat proteins were translated and processed (by the RNA-1-encoded 24K protease) as efficiently as seen in the presence of replication.

To assess the RNA content within the particles, the same samples of purified particles as the ones analyzed in Fig. 3a (lanes 1 to 4) were electrophoresed on a nondenaturing agarose gel to assess the protein and RNA contents of assembled particles (29, 30) (Fig. 3b). All four samples gave similar patterns of bands (in terms of the size and intensity of bands) when the gel was stained for protein (Fig. 3b, left). The multiple bands are due to the presence or absence of the above-mentioned 24 amino acids at the C terminus of the S coat protein that leads to an overall difference in the surface charge of the particles and hence affects their mobility during electrophoresis (30). In contrast, only those particles produced in the presence of full-length, replication-competent RNA-1 (Fig. 3, lanes 1 and 2) contained detectable nucleic acid, as judged by ethidium bromide staining (Fig. 3b, right); those produced in the presence of RNA-1-32E (lanes 3 and 4) appeared to be devoid of RNA. Indeed, no RNA could be extracted from either of the samples produced from coinfiltration of pBinP32E.

The presence of correctly processed L and S proteins in particles produced in the presence of RNA-1-32E confirms that this RNA is translationally active and produces sufficient 24K protease to process the RNA-2-derived coat protein precursors, thereby generating particles (Fig. 3a, lanes 3 and 4). However, the 32E mutation not only abolished packaging of RNA-1-32E but also abrogated packaging of coexpressed RNA-2, an RNA which is efficiently packaged in the presence of replication-competent RNA-1 (Fig. 3, lanes 1 and 2). Although it is possible that deletion of the 5′ UTR in RNA-1-32E could remove a recognition sequence vital for the incorporation of RNA-1 into capsids, the absence of such a recognition sequence cannot explain the lack of encapsidation of full-length RNA-2. The most likely explanation is that the inability of RNA-1-32E to support replication is responsible for the lack of packaging of both RNAs.

**FIG 4:**
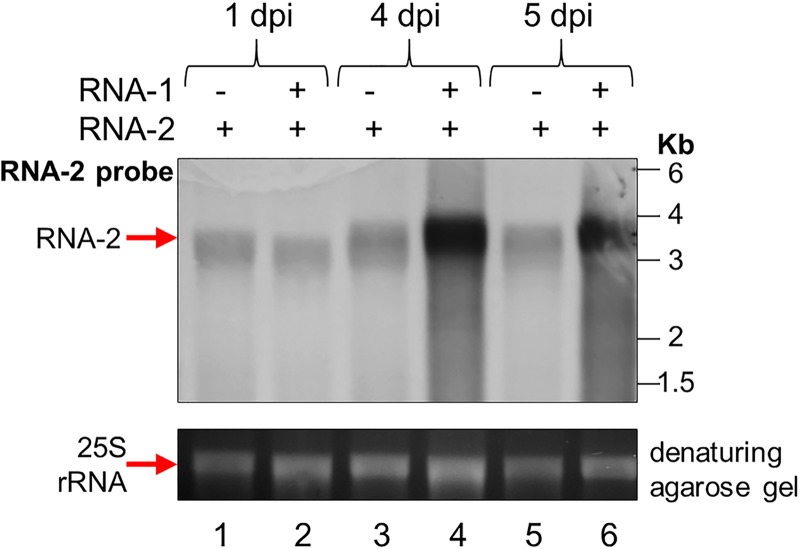
RNA-2 is abundant in the cell even in the absence of RNA-1. (Top) Northern blot of total RNA extracted from agroinfiltrated leaf material probed with an RNA probe specific for RNA-2 positive strands. Leaves were either agroinfiltrated with pEAQ-RNA-2 alone or coinfiltrated with both pBinPS1NT and pEAQ-RNA-2 and were harvested at 1, 4, and 5 days postinfiltration (dpi). (Bottom) Ethidium bromide-stained denaturing agarose gel prior to transfer of the RNAs to the membrane, showing the levels of 25S rRNA present in each sample as a loading control.

A possible, trivial, explanation for the apparent link between replication and encapsidation described above is that, in the absence of replication, the transcript-derived RNA molecules are degraded; thus, insufficient copies of full-length viral RNA molecules accumulate within cells for packaging to be detected by the assays used. To investigate this possibility, leaves were infiltrated with pEAQ-RNA-2 (containing the sequences of both wt RNA-2 and P19), in the presence or absence of pBinPS1NT. Northern blot analysis of RNA extracted from the leaves at 1, 4, and 5 days postinfiltration (dpi) showed the clear presence of full-length RNA-2 at all times, even in the absence of RNA-1 (Fig. 4). It seems unlikely that these levels of full-length RNA-2 molecules would escape detection in particle preparations if they were packaged. Thus, the failure to detect any RNA in particles from leaves infiltrated with pBinP32E and pBinPS2NT is not due to the absence of sufficient full-length RNA within the plant cells. However, the presence of RNA-1 leads to increased RNA-2 levels at later time points (4 dpi onwards), as would be expected when replication is taking place.

### Creation of mutants in the 87K replicase.

The above-described results strongly suggest that a replication-competent version of RNA-1 is required for encapsidation of both RNA-1 and RNA-2 but do not provide evidence for the direct involvement of the RNA-1-encoded replication-associated proteins in the process. To investigate this, point mutants within the region of RNA-1 encoding the 87K replicase were generated to create a replication-deficient version of RNA-1 with minimal sequence changes. The 87K replicase protein of RNA-1 contains a GDD motif, a motif that is highly conserved across numerous viral RNA polymerases and is thought to be critical for replicase function through its interaction with divalent cations (31). Thus, point mutations in this site are predicted to debilitate or eliminate replicase activity (32).

Initial attempts to create mutants in the GDD motif using pBinPS1NT were unsuccessful, as plasmids containing full-length RNA-1 are unstable in Escherichia coli and suffer mutations and sequence rearrangements (33). To alleviate this problem, a new infectious clone of RNA-1 was designed (named pEAQ-RNA-1-Int), which contains four introns within the coding region in order to prevent the expression of potentially toxic RNA-1-derived sequences in bacteria (34, 35). The introns were taken from the PSK7 gene of *Petunia* × *hybrida* (GenBank accession number AJ224165.1), and the four introns that were chosen would introduce frameshift mutations into the coding sequence of RNA-1 if they were not correctly spliced; the insertion sites were chosen as described previously (34), with their positions shown in Fig. 2. This new intron-containing version of RNA-1 (RNA-1-Int) was synthesized by GeneArt and inserted into the pEAQ vector. This new sequence proved to be substantially more stable in E. coli than its non-intron-containing counterpart, allowing two mutants to be generated through the mutation of 1 or 3 bases, respectively. In the first mutant, the GDD motif was mutated to GAD (pEAQ-RNA-1-Int-GAD), while in the second, it was mutated to AAA (pEAQ-RNA-1-Int-AAA).

**FIG 5:**
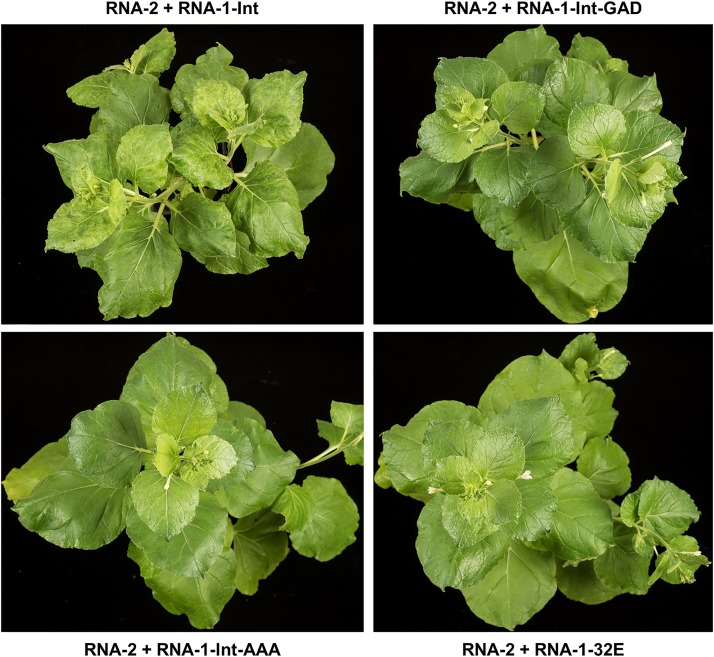
GDD mutants of RNA-1 abolish viral symptoms. Agroinfiltration was used to coexpress pEAQ-RNA-2 and an RNA-1-based construct, as indicated. Photographs were taken from above at 30 days postinfiltration to show any symptoms on the upper, systemic leaves. RNA-1-Int (expressing a wild-type replicase) causes systemic symptoms in upper leaves of N. benthamiana when coexpressed with RNA-2, as seen by mottled yellowing and curling of the upper leaves (top left-hand panel). In contrast, replicase mutants RNA-1-Int-GAD and RNA-1-Int-AAA, just like RNA-1-32E, do not cause symptoms when coexpressed with RNA-2.

To ascertain the biological activity of these mutant RNA-1 constructs, they were agroinfiltrated into N. benthamiana plants in the presence of pEAQ-RNA-2. After 30 days, the only plants to show symptoms characteristic of a systemic CPMV infection (curling and mottled yellowing of the upper leaves) were those coinfiltrated with pEAQ-RNA-1-Int and pEAQ-RNA-2 (Fig. 5), while the plants infiltrated with pEAQ-RNA-1-Int-GAD or pEAQ-RNA-1-Int-AAA in the presence of pEAQ-RNA-2 were symptomless, as were control plants coinfiltrated with pBinP32E and pEAQ-RNA-2. This indicates that the introns in RNA-1-Int were correctly spliced to yield a functional, infectious RNA-1 molecule, while the mutations in the GDD motif abolished symptom formation.

### Point mutations in the 87K replicase sequence affect RNA packaging.

**FIG 6:**
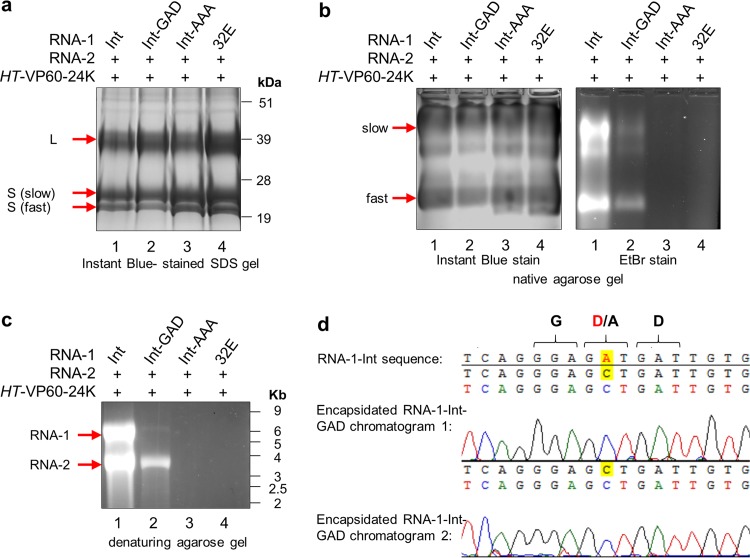
GDD mutants of RNA-1 are encapsidation deficient. Particles were purified from leaves agroinfiltrated with pEAQ-RNA-2 and an RNA-1-based construct, as indicated. The same preparation of purified particles was used for the gels shown here. (a) Protein content of particles visualized by SDS-PAGE and Instant Blue staining. The correctly processed large (L) coat protein and two electrophoretic forms of the small (S) coat protein present in all samples are indicated. (b) Purified particles from the same preparations as in panel a were analyzed in duplicate on a native agarose gel. (Left) Half of the gel was stained with Instant Blue protein stain to reveal equal loading of particles; (right) the other half of the gel was stained with ethidium bromide to visualize encapsidated nucleic acid. The positions of the L coat protein and two forms of the S coat protein are indicated to the left of the gel in panel a. Note that in panel b, CPMV particles separate into distinct electrophoretic populations based on the presence or absence of the labile 24 amino acids at the C terminus of the small coat protein (2) as seen in panel a. (c) RNA extracted from equal amounts of purified particles analyzed on an ethidium bromide-stained denaturing agarose RNA gel to reveal encapsidated RNA-1 and RNA-2. (d) Nucleic acid sequencing was carried out on the encapsidated RNA-1-Int-GAD seen in lane 2 in panel c, and this revealed that the encapsidated RNA has preserved the GAD mutation and has not reverted to wild-type GDD. Duplicate sequencing chromatograms are aligned to the wild-type sequence encoding GDD to highlight the point mutation, with the relevant amino acids indicated above. Sequencing was carried out by Eurofins Scientific, and sequence alignment was carried out using Vector NTI Advance 11.5.3.

Particles were purified from agroinfiltrated leaves coexpressing pEAQ-RNA-2 and the different variants of RNA-1 as described above, and the preparations were analyzed by SDS-PAGE to examine the levels of the processed coat proteins (Fig. 6a). Furthermore, samples were analyzed on a nondenaturing agarose gel to assess the protein and RNA contents of the particles by Instant Blue and ethidium bromide staining, respectively (Fig. 6b). Staining of the SDS gel with Instant Blue revealed that similar levels of both the L and S coat proteins were present in all samples, indicating that the intron-containing RNA-1 variants are all translationally active and produced a functional 24K protease capable of cleaving the L and S proteins from their RNA-2-encoded precursor. Equal amounts of purified particles from each preparation, as judged by protein staining, were loaded onto nondenaturing agarose gels (Fig. 6b, left), and all samples had profiles of electrophoretic forms similar to that seen in Fig. 3b. However, there was a clear difference in nucleic acid contents of the particles in different preparations, as revealed by staining of the native agarose gel with ethidium bromide (Fig. 6b, right). Particles extracted from leaves infiltrated with pEAQ-RNA-1-Int-GAD (Fig. 6b, lane 2) contained less RNA than those made with pEAQ-RNA-1-Int (lane 1), while those from leaves infiltrated with pEAQ-RNA-1-Int-AAA (lane 3) or pBinP32E (lane 4) contained no detectable nucleic acid.

To assess the nature of the RNAs within the purified particles, equal amounts of purified particles from each sample were treated with micrococcal nuclease to digest any residual nonencapsidated nucleic acid, and equal volumes of extracted RNA preparations were analyzed by denaturing agarose gel electrophoresis in the presence of ethidium bromide (Fig. 6c). While particles purified from plants infiltrated with pEAQ-RNA-1-Int and pEAQ-RNA-2 (Fig. 6c, lane 1) contained approximately equal amounts of RNA-1 and RNA-2, as expected from a wild-type infection, particles purified from plants infiltrated with RNA-1-Int-GAD and pEAQ-RNA-2 (lane 2) contained far less RNA-1 than RNA-2: the ratio was closer to 6% RNA-1 and 94% RNA-2 as measured by band densitometry in three replicates of the experiments. Moreover, the overall RNA content was clearly reduced, confirming the result shown in Fig. 6b: UV-visible (UV-Vis) spectrophotometry of RNA extracted from equal amounts of purified particles in three replicate experiments revealed that particles made with RNA-1-Int-GAD contained only about 10% of the RNA present in the equivalent number of particles (as judged by protein content) made with RNA-1-Int. Neither RNA-1 nor RNA-2 could be detected in particles generated using RNA-1-Int-AAA or RNA-1-32E (Fig. 6, lanes 3 and 4).

**FIG 7:**
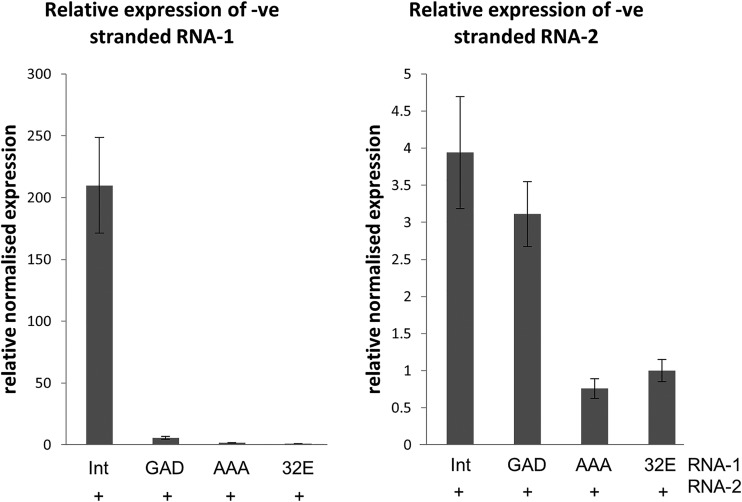
The 87K GAD mutation reduces replication efficiency, while the AAA mutation abolishes it. Total RNA was extracted from leaves agroinfiltrated with pEAQ-RNA-2 and an RNA-1-based construct, as indicated. Gene-specific qRT-PCR was carried out to quantify negative (-ve)-stranded RNA-1 and RNA-2 (replication intermediates) in the different samples. Data from three replicate experiments were analyzed using Bio-Rad CFX software to show normalized expression of negative-stranded RNA-1 (left) and RNA-2 (right) relative to RNA-1-32E. Error bars represent standard errors of the means.

The fact that the GAD single point mutation in the viral replicase has such a negative impact on RNA encapsidation, and the fact that it alters the ratio of RNA-1 to RNA-2 within the particles, is consistent with a link between encapsidation and replication and suggests that this mutation reduces, but does not abolish, the activity of the 87K replicase. Sequence analysis of the encapsidated RNA-1 revealed that it still carried the GAD mutation, with no sign of a reversion to GDD (Fig. 6d). To demonstrate that mutations in the GDD motif directly affect viral replication, quantitative real-time reverse transcriptase-PCR (qRT-PCR) was carried out to assess the level of negative-sense viral RNA in agroinfiltrated leaves, since these are replication intermediates and are not produced by transient expression itself. This is important since the pEAQ expression system used to express all the constructs is specifically designed to direct very high levels of transcription from agrobacterial transfer DNA (T-DNA), and the transcripts are indistinguishable from the positive-sense products of replication. In contrast, the generation of negative-stranded RNA is specific to RNA-dependent RNA replication. To quantify negative-stranded viral RNA, cDNA was generated from total leaf RNA extracted from the agroinfiltrated leaves with forward primers (binding to negative-stranded RNA) specific for the target sequences (RNA-1 or RNA-2) and reverse primers (binding to the messenger-sense RNA) for the reference genes. qRT-PCR analysis was then carried out on this population of cDNA, and the amounts of RNA-1 or RNA-2 negative strands were normalized to the amounts quantified in leaves expressing RNA-1-32E (Fig. 7), which has been previously shown to be replication incompetent (28). The results indicate that, compared to leaves infiltrated with pEAQ-RNA-1-Int, leaves infiltrated with pEAQ-RNA-1-Int-GAD had a 37-fold reduction in the levels of RNA-1 and a 1.3-fold reduction in the levels of RNA-2 negative strands. In contrast, RNA-1-Int-AAA did not produce any RNA-1 or RNA-2 negative strands above background levels (i.e., amounts quantified in samples containing RNA-1-32E). These results confirm that the GAD mutation reduces but does not eliminate RNA replication, while the AAA mutation abolishes it. Moreover, the disproportionate effect on the replication of RNA-1 compared with RNA-2 is consistent with the higher levels of RNA-2 found in particles (Fig. 6C, lane 2). Taken together, these data provide strong evidence that a reduction in replication efficiency causes a concomitant reduction in RNA packaging and confirms the critical role of the GDD motif in the replicase function of the CPMV 87K protein.

### Encapsidation of RNA-2 is linked to its efficiency of replication.

**FIG 8:**
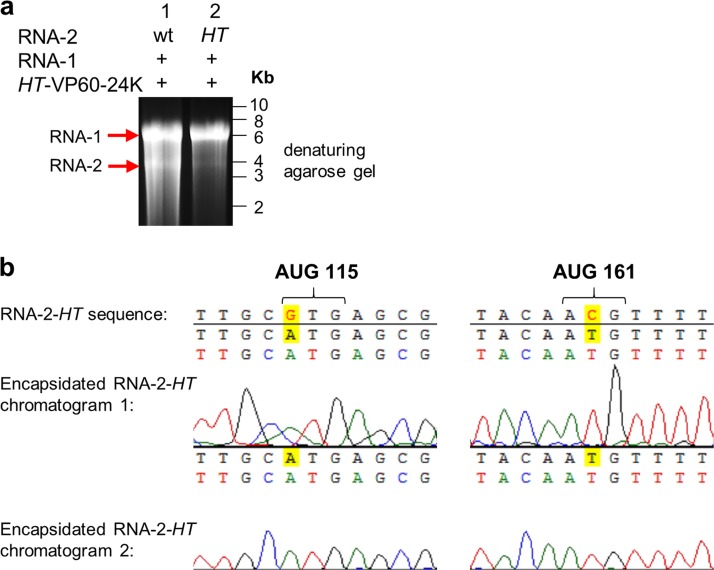
RNA-2-*HT* is encapsidated and reverts to the wild type. (a) RNA from purified particles electrophoresed on an ethidium bromide-stained denaturing agarose gel. Particles were purified from leaves after agroinfiltration with pBinPS1NT, together with pEAQ-*HT*-VP60-24K and an RNA-2 construct, as indicated. (b) Sequence analysis of the RNA-2 5′ UTR at the positions of the *HT* mutations. Sequencing was performed on RNA-2 extracted from particles from leaves coinfiltrated with pBinPS1NT, pEAQ-*HT*-VP60-24K, and pEAQ-RNA-2-*HT* and shows that the *HT* mutations have reverted to the wild type prior to encapsidation.

To determine whether the ability to be replicated is essential for RNA-2 to be encapsidated, the AUGs at positions 115 and 161 were mutated to GUG and ACG, respectively, mutations collectively known as the “hypertranslatable” (*HT*) mutations (34, 36), since elimination of these AUGs had previously been shown to greatly reduce RNA-2 replication (37, 38). Plasmid pEAQ-RNA-2 (containing the sequence of wild-type RNA-2) or pEAQ-RNA-2-*HT* (with the AUG codons eliminated) was coexpressed with pBinPS1NT for provision of RNA-1 and pEAQ-*HT*-VP60-24K to guarantee consistent quantities of processed capsid protein in both samples. RNA was extracted from particles purified from the infiltrated leaves, and equal amounts of nucleic acid were separated on a denaturing agarose gel (Fig. 8). Bands at the expected size for RNA-1 (∼5.9 kbp) confirmed that the particles from both samples are encapsidation competent. However, the intensity of the band corresponding to RNA-2 (∼3.5 kbp), while clearly visible in the sample of particles produced in the presence of wild-type RNA-2, was only just visible in the RNA-2-*HT* sample (Fig. 8). This shows that the *HT* mutation decreases the encapsidation efficiency of RNA-2 but does not abolish it (Fig. 8, lane 2). Sequence analysis of the encapsidated RNA-2 from the RNA-2-*HT* sample revealed that the *HT* mutations had reverted to the wild-type sequence (Fig. 8b). This is an indication that RNA-2-*HT* retains some ability to replicate, because reversion of the *HT* mutation can occur only if viral RNA replication is taking place. The fact that revertant RNA-2 seems to be encapsidated while RNA-2-*HT* is not is further evidence that packaging of viral RNA is dependent on replication, since the *HT* mutation is known to adversely affect replication.

### Truncation and substitution of the RNA-2 coding region do not alter encapsidation.

To investigate whether the length and/or coding sequences of RNA-2 contribute to its specific encapsidation, two truncation products of wild-type RNA-2 were generated: two pairs of restriction sites were used (MfeI and XhoI) to excise a part of the RNA-2 coding sequence and religate the ends to yield XhoI-RNA-2 (∼3.3 kbp) and MfeI-RNA-2 (1.5 kbp). In addition to this, a construct (green fluorescent protein [GFP]) was generated by replacing the entire RNA-2 coding sequence (from the AUG at position 512) with the coding sequence for GFP (Fig. 2), leaving only the 5′ and 3′ UTRs from RNA-2. In all cases, the frame relationships between the main initiation codon and the upstream AUGs at positions 115 and 161 were preserved, a feature known to be important for RNA-2 replication (37–39).

**FIG 9:**
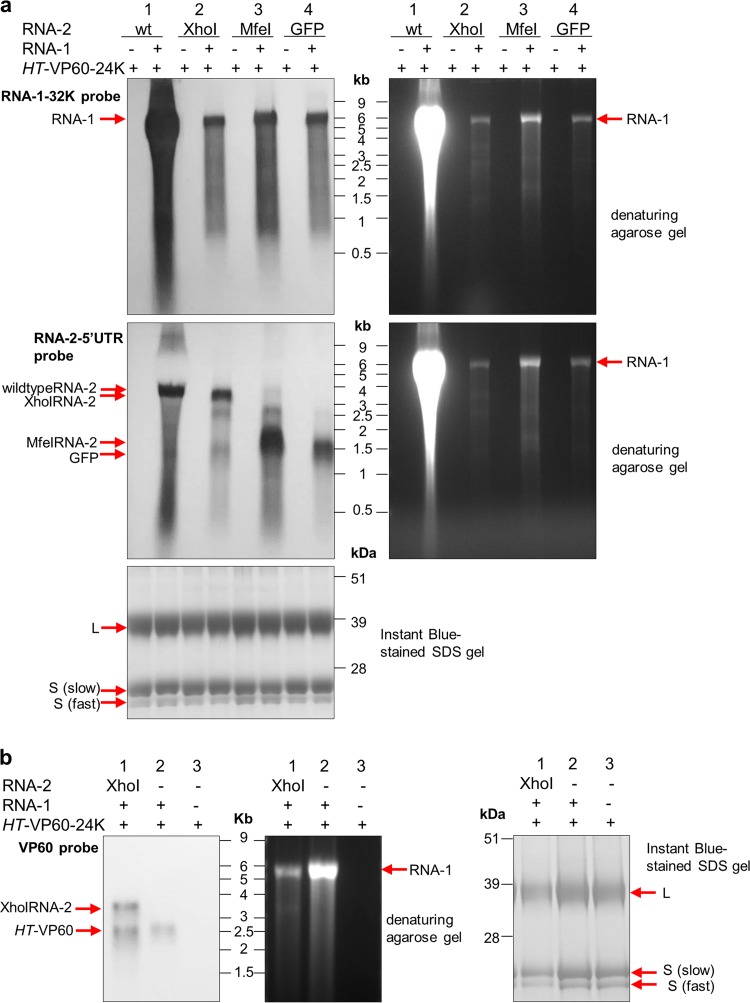
Truncated RNA-2 constructs can be encapsidated in the presence of RNA-1. (a) Northern blots of RNA packaged in particles produced with different versions of RNA-2 with and without RNA-1. RNA was extracted from particles purified from leaves agroinfiltrated with *HT*-VP60-24K and an RNA-2 construct, as indicated, in the presence or absence of pBinPS1NT. In each case, the RNA was extracted from 3 mg purified particles, and the resulting RNA was split equally on two duplicate denaturing agarose gels for subsequent Northern blotting. (Left) Detection of RNA-1 (top) or RNA-2 (middle) with probes annealing to the 32K ProC sequence of RNA-1 or the RNA-2 5′ UTR, respectively; (right) denaturing agarose gels before transfer to nylon membranes; (bottom) purified particle preparations visualized on an Instant Blue-stained denaturing SDS-PAGE gel serving as a control for processing of VP60 and the use of equal amounts of particles for each RNA extraction. (b) *HT*-VP60 is encapsidated in the presence of RNA-1. The presence of encapsidated RNA was analyzed in purified particles extracted from plants transiently expressing pEAQ-*HT*-VP60-24K with or without pEAQ-XhoI-RNA-2 and with or without pBinPS1NT. RNA was extracted from 2 mg purified particles, and the resulting RNA was loaded onto a denaturing agarose gel for subsequent Northern blotting. (Left) Immunoblot detection of VP60 with the probe annealing to a sequence within the VP60 coding region that is partially removed in the construct resulting from XhoI digestion; (middle) denaturing agarose gel before transfer to a nylon membrane; (right) Instant Blue-stained protein on a denaturing SDS-PAGE gel serving as a control for processing of the VP60 protein precursor and the use of equal amounts of particles in each RNA extraction.

Because these truncation and substitution mutants cannot produce the coat protein precursor, their capacity to be encapsidated was tested by coexpressing them with pEAQ-*HT*-VP60-24K in the presence or absence of RNA-1 (Fig. 9). Following purification of any particles produced in infiltrated leaves, RNA was extracted from equal numbers of particles (as judged by protein content), electrophoresed on denaturing agarose gels, and transferred to nylon membranes for Northern blot analysis. After transfer, one duplicate membrane was treated with a probe annealing to the 5′ UTR of RNA-2 to visualize all encapsidated RNA-2-based molecules, and the other duplicate was treated with a probe annealing to the 32K protease cofactor region to visualize RNA-1 (Fig. 9a). Samples without RNA-1 did not show any sign of either RNA-1 or RNA-2 encapsidation, which confirms our previous finding that replication is required for encapsidation. RNA-1 was encapsidated to roughly equal amounts in the samples from coexpression with the RNA-2 truncation or substitution products, while a significantly larger amount was encapsidated in the control sample coexpressed with wild-type RNA-2. This is probably due to the presence of an intact movement protein in the polyprotein encoded by the wild-type but not the variant RNA-2 molecules (Fig. 2). This enables the viral RNAs to spread from the initially infected cells, thereby reaching higher titers.

The bands corresponding to RNA-2 truncation products at ∼3.3 kbp (XhoI) and ∼1.5 kbp (MfeI) and GFP (∼1.5 kbp) were of an intensity similar to those of the wild-type RNA-2 band in the positive control (Fig. 9). Thus, truncation or even full substitution of the RNA-2 coding region does not prevent encapsidation in the presence of viral replication. It was noted that in all lanes in which RNA-1 was coexpressed, the RNA-2 5′-UTR probe detects bands at ∼2.5 and ∼1.3 kb, which correspond to the sizes of *HT*-VP60 (∼2.5 kbp) and *HT*-24K (∼1.3 kbp) from the *HT*-VP60-24K construct, suggesting that these RNAs are also encapsidated to a minor extent. This low-level encapsidation is unsurprising, since Fig. 8 and 9 show that the *HT* mutations do not abolish encapsidation and that the coding region is irrelevant. Northern blotting experiments confirmed the identities of these RNAs (Fig. 9b).

**FIG 10:**
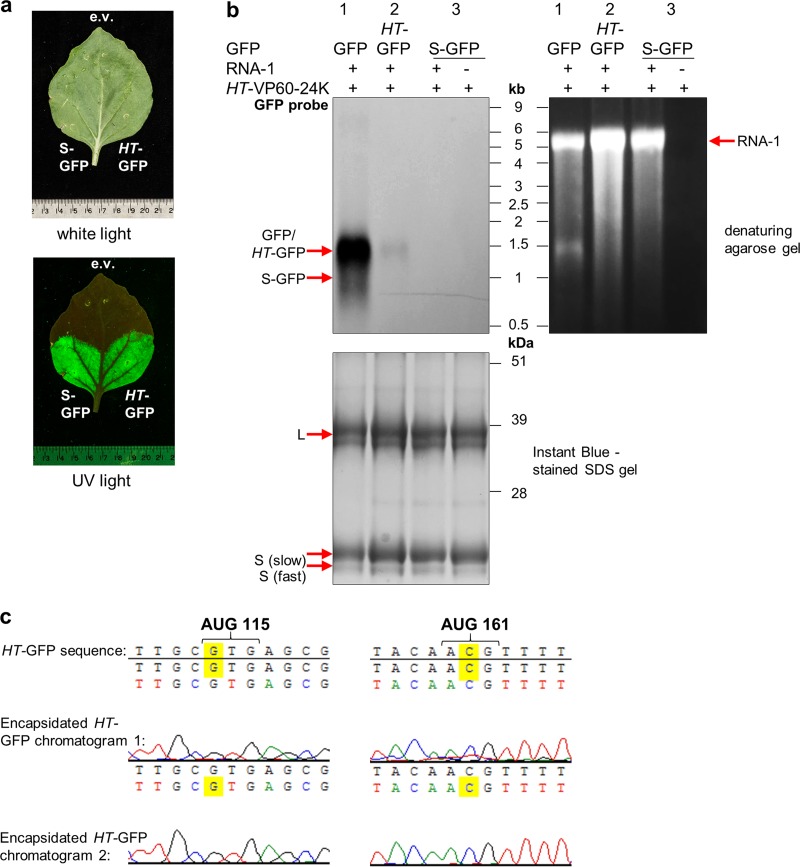
Heterologous RNA can be encapsidated when bordered by RNA-2 UTRs. (a) Expression of GFP following agroinfiltration of N. benthamiana leaves with the constructs indicated. GFP fluorescence was visualized under UV light at 7 dpi, with the empty pEAQ vector (e.v.) agroinfiltrated as a negative control. (b) RNA extracted from particles purified from plants agroinfiltrated with pEAQ-HT-VP60-24K together with GFP constructs flanked by different 5′ UTRs, as indicated, in the presence or absence of pBinPS1NT. In each case, RNA was extracted from 1.5 mg purified particles, and the resulting RNA was analyzed by denaturing agarose gel electrophoresis and subsequent Northern blotting. (Top left) Northern blotting using a probe specific for the coding region of GFP; (top right) agarose gel before transfer to nylon membranes stained with ethidium bromide; (bottom) Instant Blue-stained SDS-PAGE gel used to reveal processing of VP60 and protein content of the particle preparations. (c) Sequence analysis of the RNA-2 5′ UTR at the positions of the *HT* mutations. Sequencing was performed on *HT*-GFP RNA shown in lane 2 in panel b, from particles from leaves coinfiltrated with pBinPS1NT, pEAQ-HT-VP60-24K, and pEAQ-HT-GFP. The duplicate chromatograms show that the *HT* mutations have not reverted to the wild type prior to encapsidation and are still *HT*.

To confirm that replication is essential for the encapsidation of RNA-2 molecules harboring GFP, two additional constructs were generated in which the wild-type 5′ UTR was replaced with either a version containing the *HT* mutations (*HT*-GFP) or an entirely synthetic 5′ UTR (S-GFP), a 64-nucleotide sequence designed to be an efficient translational enhancer but without any sequence similarity to CPMV RNA-2 or any other viral RNA (H. Peyret, J. Brown, and G. P. Lomonossoff, unpublished data). The expression of S-GFP was verified by *Agrobacterium*-mediated transient expression in N. benthamiana and visualization of GFP fluorescence in the infiltrated leaves under UV light (Fig. 10a), which confirmed GFP expression and, therefore, RNA presence. The GFP constructs with different 5′ UTRs (Fig. 10b) were coexpressed with pEAQ-*HT*-VP60-24K and pBinPS1NT, and RNA extracted from equal numbers of purified particles was subjected to Northern blot analysis using a probe specific for part of the coding region of GFP. Figure 10b shows bands corresponding to the *HT*-GFP and GFP constructs (∼1.5 kbp), with the *HT* construct being encapsidated to a much lesser extent than the construct with a wild-type 5′ UTR. No signal was seen at the position of S-GFP (∼1 kbp), regardless of whether the particles were produced with or without RNA-1. The gel prior to transfer shows that RNA at a size corresponding to RNA-1 was present in all three samples in which pBinPS1NT was coinfiltrated, confirming the occurrence of viral replication and the production of encapsidation-competent particles in these samples. Because leaves expressing S-GFP show GFP fluorescence (Fig. 10a), the lack of encapsidation of S-GFP cannot be due to the lack of the presence of this RNA within cells but must instead be due to a lack of replication. Sequence analysis showed that the *HT* mutations in *HT*-GFP RNA extracted from purified particles did not revert to the wild type (Fig. 10c), unlike the situation found with RNA-2-*HT* (Fig. 8B), a difference which can be linked to the absence of the movement protein, limiting possible reversion events to the cell in which they occur.

## DISCUSSION

The work presented here establishes the linkage between the processes of viral replication and RNA packaging in CPMV and, by extension, other members of the order *Picornavirales*. In cases where the ability of RNA-1 to replicate both itself and RNA-2 was eliminated (RNA-1-32E and RNA-1-Int-AAA), no RNA was detected within particles. In contrast, a mutation that reduced but did not abolish the replication of RNA-1 (RNA-1-Int-GAD) concomitantly reduced the amount of RNA encapsidated. Similarly, the *HT* mutations in the 5′ UTR of RNA-2 were found to knock down, but not abolish, replication of RNA-2, with a parallel knockdown effect on encapsidation. Finally, it was found that truncations within the coding region of RNA-2, and, indeed, even the complete substitution of the RNA-2 open reading frame, did not abolish packaging. Taken together, these data demonstrate that packaging of both RNA-1 and RNA-2 requires replication-competent RNA-1 and that the 5′ UTRs of both RNA-1 and RNA-2 play a crucial role in replication and packaging.

The phenotype of RNA-1-Int-GAD was unexpected, as previous work with other RNA viruses, including other *Picornavirales*, suggested that the aspartate residues of the GDD motif are usually essential for replicase function, and only mutations in the glycine residue are typically tolerated (32, 40–42). However, one notable exception is the replicase of encephalomyocarditis virus, a pig-infecting cardiovirus, which still retains 3% of the replicase activity if the first aspartate residue is mutated (43); CPMV appears to be a similar case to this. It is perhaps even more surprising that the effect of the GAD mutation seems to affect replication and encapsidation of RNA-1 disproportionately more than replication and encapsidation of RNA-2 (Fig. 6 and 7). Why this might be the case is unclear, but this unequal effect was consistent over three repeats of the experiment and is seen when analyzing both encapsidation (Fig. 6) and replication (Fig. 7). The greatly reduced levels of encapsidated RNA-1 in particle preparations is presumably the cause of the lack of systemic movement (and therefore symptoms) seen with RNA-1-Int-GAD (Fig. 5).

The sequencing result on encapsidated RNA-1-Int-GAD was initially somewhat surprising, since mutations that debilitate but do not abolish replication often revert very quickly in plant RNA viruses (as indeed was the case with RNA-2-*HT*). In this case, it seems likely that low-level viral replication will lead to a low level of reversion to the wild-type sequence. This would result in the expression of a fully functional revertant 87K replicase, but the revertant RNA-1-Int RNA itself would have no selective advantage over RNA still carrying the GAD mutation in terms of its ability to be replicated. It would therefore remain at a constant low level within the RNA population of the cell and would not reach levels detectable by the methods used in this study. Nevertheless, the fact that only replicating RNA can be packaged provides a means for ensuring that only viral RNAs are found in particles, which explains why CPMV capsids produced in the absence of replication appear to be devoid of any nucleic acid (2, 23; this study). This contrasts with the situation found with other viral capsids expressed in N. benthamiana, such as turnip crinkle virus (TCV), where, in the absence of the authentic genomic RNA, significant quantities of host RNA are encapsidated (18). Particularly intriguing is the fact that RNA-2 molecules produced by transcription in the nucleus after agroinfiltration are not encapsidated despite accumulating to significant levels, being efficiently translated to yield the coat protein precursors and being replicated in the presence of a replication-competent RNA-1. These observations suggest that there is something different about the RNA molecules produced by replication compared with transcription.

One obvious difference between the RNA molecules produced by replication and those produced by transcription is the location of their synthesis (cytoplasm versus nucleus). It is possible that the colocalization of newly replicated RNA molecules and newly translated coat protein molecules in the cytopathological structures associated with virus infection (44, 45) simply means that replicated viral RNA is the only RNA available for incorporation into the nascent capsids. However, this alone does not explain why, in the absence of replication, no nucleic acids are incorporated into capsids produced by transient expression. Other features, therefore, must also play a role. For example, RNA produced via replication has a 5′-terminal genome-linked viral protein (VPg) as opposed to a cap structure found on RNA molecules generated by transcription. Thus, the presence of VPg could, in theory, provide a means of discriminating between replicated RNA and that produced by transcription. VPg is believed to arise from the cleavage of an RNA-1-encoded 112K precursor (46), which also contains the sequences of the 24K protease and the 87K replicase. The 112K protein is analogous to 3BCD from poliovirus (5), which contains the sequence of VPg (3B), the viral protease (3C), and the viral polymerase (3D). In the case of poliovirus, it has been found that 3CD can stimulate the encapsidation of VPg-linked RNA, but not transcripts lacking VPg, into particles (47, 48). This has been interpreted as indicating that there is an affinity between 3CD and VPg. If CPMV VPg is required for RNA encapsidation, its mere presence is clearly not sufficient. Indeed, we have shown that the RNA-1 polyprotein is translated in our RNA-1 mutants, which indicates that VPg must be produced as well, but it is clearly not capable of directing encapsidation when replication is not taking place. It seems likely that any role that VPg plays in packaging is dependent on its linkage to the viral RNAs, which takes place only during replication. A further possibility is that nascent positive strands produced via replication fold into the type of hairpin structures identified as being important for packaging in human parechovirus (8), while full-length RNA molecules produced by transcription in the nucleus and then exported to the cytoplasm adopt assembly-incompetent structures. However, the fact that CPMV efficiently encapsidates two RNAs of different sizes that lack any sequence homology apart from at the extreme 5′ and 3′ ends makes the idea that the recognition of packaging signals is responsible for their specific packaging unlikely.

Whatever its mechanism, the coupling of RNA replication to encapsidation provides a route to the selective packaging of viral RNAs in the *Picornavirales*. Moreover, the observation that only replication-competent versions of CPMV RNA-2 in which the open reading frame has been replaced with GFP can be packaged suggests that the linkage could be used to specifically incorporate heterologous RNAs expressed in plant cells within particles. The ability to efficiently encapsidate specific plant-expressed RNAs has profound implications for the future development of CPMV for bionanotechnological applications.

## MATERIALS AND METHODS

### Genomic CPMV constructs.

Plasmids pBinPS1NT (27) and pBinP32E, harboring a deleted 5′ UTR (28), were used to produce replication-competent and incompetent versions of CPMV RNA-1, respectively. A new full-length version of RNA-1, suitable for the generation of point mutants, was designed by inserting the sequences of introns 2, 3, 4, and 7 of the *Petunia* × *hybrida* PSK7 gene (GenBank accession number AJ224165.1), according to a previously described protocol (34), after the guanosines at positions 3082, 4301, 5277, and 5430, respectively, of the RNA-1 sequence (GenBank accession number X00206.1) (36). The intron-containing RNA-1 sequence was placed between the cauliflower mosaic virus (CaMV) 35S promoter and the *Agrobacterium tumefaciens* nopaline synthase (nos) terminator sequences used in the pEAQ-*HT* expression vector (GenBank accession number GQ497234.1) (14), flanked by PacI and AscI restriction sites on the 5′ and 3′ ends, respectively, and synthesized by GeneArt (Life Technologies). The plasmid carrying the synthesized construct (pMA-RNA1-Int) was used as a template for site-directed mutagenesis using the GeneArt site-directed mutagenesis system (Life Technologies). To make RNA1-Int-GAD, the DNA sequence encoding the GDD motif (GGAGATGAT) was mutated to GGAGCTGAT to change the amino acid sequence to GAD. To make RNA-1-Int-AAA, this first mutant was used as a template for a second round of site-directed mutagenesis to change the DNA sequence to GCAGCTGCT, which changes the amino acid sequence to AAA. The three versions of the intron-containing RNA-1 were then cloned into the pEAQ-*HT* binary vector using the PacI and AscI restriction sites, which yielded three expression vectors, namely, pEAQ-RNA1-Int, pEAQ-RNA1-Int-GAD, and pEAQ-RNA1-Int-AAA. Note that these pEAQ plasmids also include a P19 silencing suppressor expression cassette in the T-DNA alongside the RNA-1 expression cassette.

Full-length wild-type CPMV RNA-2 was expressed from either pBinPS2NT (27) or pEAQ-RNA-2 (2). When pBinPS2NT was used, leaves were coinfiltrated with pBIN61-P19 to supply the P19 suppressor of gene silencing (49). This was not necessary when pEAQ-RNA-2 was used, since this plasmid already includes a P19 expression cassette on the T-DNA alongside RNA-2. XhoI-RNA-2 and MfeI-RNA-2 were expressed from pEAQ-XhoI-RNA-2 and pEAQ-MfeI-RNA-2, respectively, which were generated by XhoI or MfeI digestion and subsequent religation of pEAQ-RNA-2. Plasmid pEAQ-RNA-2-*HT*, directing the expression of RNA-2-*HT*, was created by amplifying the coding sequence of RNA-2 starting from the AUG at position 512; this sequence was inserted into pEAQ-*HT* using the restriction sites AgeI and StuI in its polylinker, which places the coding region between the CPMV RNA-2 UTRs with the *HT* mutations (mutations of the AUG codons in the 5′ UTR at positions 115 and 161) in the 5′ UTR (14). GFP was expressed with the wild-type RNA-2 UTRs using pEAQ-GFP, which was generated by cloning the entire GFP expression cassette from pM81-FSC2-GFP (49) into pEAQ-*HT* using PacI and AscI restriction ligation. GFP with the *HT* UTRs was expressed from pEAQ-*HT*-GFP (14). GFP with a synthetic 5′ UTR (S-GFP) was expressed from pEAQ-S-GFP, generated by replacing the 5′ UTR upstream of the GFP coding region in pEAQ-*HT*-GFP by the 5S0 sequence, a synthetically generated 64-nucleotide sequence designed to act as a translational enhancer but with no sequence homology to any known 5′ UTR. This sequence will be discussed in more detail elsewhere (Peyret et al., unpublished), but its sequence is TTTAAGAGACGCAACCACAACGCTCTAACGCAATCAATCTACATTATATTAAACGTCTCTAAAA.

To provide a source of the CPMV capsid proteins in the absence of or in addition to RNA-2, pEAQ-*HT*-VP60 (23) or pEAQexpress-VP60-24K (50) was used to express *HT*-VP60 or *HT*-VP60-24K, respectively. These plasmids supply the coat protein precursor and, in the case of pEAQexpress-VP60-24K, also supply the 24K protease, which is necessary for processing of VP60 and particle formation. Both plasmids also carry an expression cassette for P19 on the same T-DNA as the expression cassette(s) for VP60 and/or 24K. In all cases, when a DNA construct is being referred to, its full name, including the name of the plasmid, is given. When transcribed RNA is discussed, the designation of the plasmid is omitted.

### Expression in plants.

All gene constructs were cloned and maintained in E. coli one shot TOP10 cells (Life Technologies) grown at 37°C in LB medium containing 50 μg/ml kanamycin for plasmid selection. For intron-containing RNA-1 constructs, E. coli cells were grown at 28°C in LB medium containing kanamycin and glucose (2%, wt/vol). Colonies were verified by colony PCR and sequence analysis (Eurofins Scientific). Plasmids having the correct sequence were transformed into electrocompetent Agrobacterium tumefaciens strain LBA4404 and used for transient expression in Nicotiana benthamiana plants via agroinfiltration using a needleless syringe as described previously (51). Plants were grown in greenhouses maintained at 25°C with supplemental lighting to provide 16 h of daylight. Infiltrated leaves were harvested at 6 to 7 days postinfiltration (dpi).

### Extraction, purification, and characterization of CPMV particles.

Particles were purified from infiltrated leaves by polyethylene glycol (PEG) precipitation and pelleting as described previously (29). The protein content of the preparations was determined by electrophoresis of denatured samples on 12% (wt/vol) Bis-Tris NuPAGE gels (Life Technologies) and by using the Pierce bicinchoninic acid (BCA) protein assay kit microplate protocol (Thermo Fisher Scientific). After electrophoresis, proteins were detected by staining with Instant Blue (Expedeon). Equal amounts of purified particles for each sample (as determined by protein content) were also analyzed by electrophoresis on nondenaturing 1.0 or 1.2% (wt/vol) agarose gels (52). Sets of samples were electrophoresed in duplicate on the same agarose gel, which was sliced into duplicate halves after electrophoresis. One half of the gel was stained to reveal protein, while the other half of the gel was stained with ethidium bromide to reveal nucleic acid.

### Encapsidated RNA extraction and purification.

RNA was extracted from equal amounts of purified particles (as determined by protein contents of purified particle preparations). The purified particles were first treated with micrococcal nuclease (NEB) (4 μl of enzyme in 50-μl reaction mixtures) at 37°C for 15 min. The reaction was terminated by adding NETS buffer (100 mM NaCl, 10 mM Tris-HCl [pH 7.5], 2% [wt/vol] SDS, 1 mM EDTA [final concentration]) supplemented with EGTA (20 mM final concentration). The nuclease-treated particles were then heated to 65°C for 10 min, and the nucleic acid was extracted by phenol-chloroform extraction as previously described (53). RNA was recovered after lithium chloride precipitation: 8 M lithium chloride solution (Sigma-Aldrich) was added to the aqueous fractions to a final concentration of 2 M, and the samples were kept at 4°C for 1 to 2 h and then at −20°C overnight. The samples were then centrifuged at 16,000 × *g* for 15 to 30 min at 8°C to recover the RNA pellets. The pellets were washed with 70% (vol/vol) ice-cold ethanol and centrifuged again for 10 min, the ethanol wash was removed, and the pellets were allowed to air dry before being resuspended in a small volume of sterile nuclease-free water.

### Analysis of RNA.

RNA extracted from equal amounts of particles (as measured by protein content) was analyzed by electrophoresis under denaturing conditions in formaldehyde-containing agarose gels, in the presence of ethidium bromide (54). For Northern blotting, the RNA was transferred from the agarose gel to a positively charged nylon membrane and probed with digoxigenin (DIG)-labeled RNA probes specific for the sequence of interest. The probe was synthesized from a PCR fragment using the DIG starter labeling kit (Roche) and was detected on the Northern blot membrane by alkaline phosphatase-conjugated anti-DIG antibody (Roche). The signal was detected colorimetrically using BCIP (5-bromo-4-chloro-3-indolylphosphate) substrate tablets (Sigma-Aldrich). For sequencing, purified RNA samples were further treated with Turbo DNase (Thermo Fisher Scientific) using the “thorough treatment” protocol recommended by the manufacturer and then used for gene-specific reverse transcriptase PCR with the SuperScript III one-step RT-PCR system with Platinum *Taq* high-fidelity DNA polymerase (Life Technologies). The reverse-transcribed and amplified DNA thus generated was sequenced (Eurofins Scientific), and the resulting sequences were analyzed using Vector NTI Advance 11.5.3.

For qRT-PCR, total leaf RNA from agroinfiltrated leaves from three experimental repeats was purified using the Qiagen RNeasy plant minikit (including the DNase I digestion step) and then further treated with Turbo DNase (Thermo Fisher Scientific) using the thorough treatment protocol recommended by the manufacturer. The selected reference genes were PP2A, FBox, and L23, which have been previously identified as robust reference genes for qRT-PCR in virus-infected Nicotiana benthamiana (55), along with the P19 suppressor of gene silencing, which is always expressed from pEAQ-RNA-2 and therefore behaves as the genetic background in this experiment. In order to quantify negative-stranded viral RNA, gene-specific cDNA was generated from total leaf RNA using the Superscript III cDNA synthesis supermix kit (Life Technologies) with a gene-specific primer mix. This consisted of reverse primers (binding to messenger-sense RNA) for the four reference genes and forward primers (binding to negative-sense RNA) specific for the 87K region of CPMV RNA-1 (for quantification of RNA-1) and the 3′ UTR of CPMV RNA-2 (for quantification of RNA-2). Samples of cDNA were prepared in triplicates in Go*Taq* qPCR master mix (Promega) and loaded in Thermofast white 96-well qPCR plates (Thermo Fisher Scientific). qRT-PCRs were carried out in a Bio-Rad CFX96 real-time C1000 touch thermal cycler, and Bio-Rad CFX Manager analysis software was used to automate analysis of the data using the 2^−ΔΔ^*^CT^* method, using the four reference genes described above. For both RNA-1 and RNA-2 quantification, quantification was normalized to the value obtained with the RNA-1-32E-containing sample. The efficiency of all primer pairs was analyzed using pooled cDNA from infiltrated leaf tissue to confirm that the amplification factor value for each pair was between 1.8 and 2.2. The primers used and their sequences are available upon request.

### Data availability.

The authors agree that any materials and data that are reasonably requested by others are available from a publicly accessible collection or will be made available in a timely fashion, at reasonable cost, and in limited quantities to members of the scientific community for noncommercial purposes. The authors guarantee that they have the authority to comply with this ASM policy either directly or by means of material transfer agreements.
